# Five‐year follow‐up of the risk factors for psychological distress in youth after the Great East Japan Earthquake and nuclear disaster: The Fukushima Health Management Survey

**DOI:** 10.1002/pcn5.70377

**Published:** 2026-07-15

**Authors:** Hideki Sato, Masaharu Maeda, Rie Mizuki, Naoko Horikoshi, Shuntaro Itagaki, Itaru Miura, Tetsuya Ohira, Seiji Yasumura

**Affiliations:** ^1^ Department of Disaster Psychiatry Fukushima Medical University School of Medicine Fukushima Japan; ^2^ Radiation Medical Science Center for the Fukushima Health Management Survey Fukushima Medical University Fukushima Japan; ^3^ Fukushima Mental Health and Welfare Center Fukushima Japan; ^4^ Faculty of Psychology Iryo Sosei University Fukushima Japan; ^5^ Department of Public Health Fukushima Medical University School of Medicine Fukushima Japan; ^6^ Department of Neuropsychiatry Fukushima Medical University School of Medicine Fukushima Japan; ^7^ Department of Epidemiology Fukushima Medical University School of Medicine Fukushima Japan

**Keywords:** age groups, cohort study, Great East Japan Earthquake, nuclear accident, psychological distress

## Abstract

**Aim:**

This study examined the longitudinal association between age and mental health status to identify the risk factors for severe psychological distress in youth who experienced the Great East Japan Earthquake of 2011.

**Methods:**

A two‐wave longitudinal study was conducted using data from the Mental Health and Lifestyle Survey of Japan after the event for fiscal years (FY) 2011 and 2016. Multiple logistic regression analyses were conducted using age subcohorts from the baseline, FY2011, and psychological distress from FY2016. Ultimately, 23,682 participants aged 16–24, 25–64, and ≥ 65 years (*n* = 711, 13,756, and 9215, respectively, at the FY2011 baseline) were analyzed.

**Results:**

Multiple logistic regression analyses revealed that, after adjustment for covariates, the 16–24 age range was significantly and positively associated with severe psychological distress in FY2016 compared with the ≥ 65 age range. Moreover, for the 16–24‐year age group, baseline psychological distress, post‐traumatic stress disorder (PTSD) symptoms, and bereavement were significantly correlated with severe psychological distress in FY2016.

**Conclusions:**

The findings suggested that youths showed higher rates of severe psychological distress following the Fukushima disaster. PTSD symptoms and bereavement may have been risk factors for severe psychological distress.

## INTRODUCTION

On March 11, 2011, Japan was hit by a devastating 9.0‐magnitude earthquake, known today as the Great East Japan Earthquake (GEJE). Its seismic center was in the seabed, 130 km off the Sanriku coast. The subsequent tsunami initiated a nuclear accident at Tokyo Electric Power Company's Fukushima Daiichi Nuclear Power Plant.^1^ Radiation hazards ensued, quickly forcing numerous people residing in the Fukushima prefecture to relocate to long‐term evacuation premises.^2^ In particular, the coastal area, Hamadōri, which was the location of the plant, was seriously affected. Consequently, over 160,000 residents were forced to evacuate within the first year after the accident.^3^

As of February 2024, over 26,000 people have been forced to evacuate in the Fukushima prefecture.^4^ Taking into consideration the health impact of this complex disaster, the prefectural government conducted the Mental Health and Lifestyle Survey (MHLS) as part of the Fukushima Health Management Survey to assist with the long‐term management of residents' health.^5^, ^6^ Previous studies have found that rates of severe psychological distress are elevated following natural and nuclear disasters.^7^, ^8^, ^9^, ^10^, ^11^, ^12^ The Kessler Psychological Distress Six‐Item Scale (K6) considers a score of ≥ 13 to indicate severe psychological distress.^13^ Japan's fiscal year (FY) begins in April, and the MHLS revealed that 14.6% of individuals 16 years of age or older may have experienced severe psychological distress in FY2011.^14^ Although the percentage of those with severe psychological distress is declining, more than a decade after the Fukushima disaster, it remains higher than that of the general population not affected by the disaster in Japan (3%).^8^, ^15^

Several studies have shown that natural and nuclear disasters could have a greater impact on the mental health of youth at the time of disaster. Youth aged 16–24 years are at a higher risk of mental illness than adults,^16^, ^17^ and the development of mental illness in early adolescence has a negative impact on prognosis and functioning.^18^, ^19^ When youth experience natural and radiation disasters, mental health problems are likely to become apparent due to the breakdown of social networks, failure at school, impaired or unstable employment, and poor family and social functioning.^20^, ^21^ Symptoms of post‐traumatic stress disorder (PTSD), depression, and anxiety after natural and human‐caused disasters that occur during adolescence have been known to continue for many years.^22^

Norris et al.^23^ conducted an empirical review focusing on mental health, which included over 60,000 victims from 160 disasters, including natural disasters. They observed that young age is associated with an increased impact of disasters on mental health. A recent MHLS^3^ also revealed that younger individuals experience severe psychological distress to a greater extent than older individuals, highlighting the vulnerability of youth. Hayashi et al.^21^ used FY2011 MHLS data to conduct a cross‐sectional study of risk factors associated with severe psychological distress among adolescents aged 15–19 years. Their results demonstrated that severe psychological distress is significantly correlated with the female sex, poor subjective health status, sleep insufficiency, bereavement, and risk perception of genetic effects due to radiation. However, the longitudinal association between age subcohorts and mental health status after the GEJE has not been examined.

Consequently, in this study, we aimed to examine the longitudinal risk factors correlated with severe psychological distress in youth after the GEJE and ensuing nuclear disaster, using baseline and 5‐year follow‐up data from the MHLS from FY2011 and FY2016, respectively.

## METHODS

### Study design

A two‐wave longitudinal study was conducted as part of the MHLS. This study was part of the Fukushima Health Management Survey, conducted in FY2011 (January 2012) and FY2016 (January 2017). The protocol and overview of the Fukushima Health Management Survey have been published previously.^5^, ^6^

The MHLS aimed to assess the mental health and lifestyle of those who had been displaced by the Fukushima Daiichi Nuclear Power Plant accident, prevent lifestyle‐related diseases affecting them, and provide appropriate care. The survey comprised basic assessments to estimate the levels of external radiation exposure among all 205,000 residents during the approximately 4‐month period following the Fukushima disaster. In the MHLS, self‐administered questionnaires were mailed to the target population across 12 municipalities, including the evacuation zones and radiation hotspots in Date City, as designated by the Japanese government. This occurred on January 18, 2012, approximately 10 months after the earthquake. The evacuation zones included Hirono Town, Naraha Town, Tomioka Town, Kawauchi Village, Okuma Town, Futaba Town, Namie Town, Katsurao Village, Iitate Village, Minamisoma City, Tamura City, and the Yamakiya district of Kawamata Town. When a respondent was determined to have mental and/or physical health problems, psychosocial intervention was provided by telephone by healthcare professionals, including clinical psychologists, nurses, and public health nurses, according to predetermined criteria.^5^, ^8^ If the respondent could not be reached, they were contacted by mail.

The present study was conducted using FY2011 and FY2016 data from participants aged ≥ 16 years during FY2011. The self‐administered questionnaires used in the MHLS can be found among the materials of the Fukushima Health Management Survey's investigative committee.^24^

### Study population

The study population included residents of the target areas who were ≥ 16 years old, as of April 1, 2011. In the MHLS, data on the personal information of the target population were obtained from the municipalities' 2011 resident registries, and we targeted 180,604 residents in FY2011. By October 31, 2012, 73,569 individuals had responded, representing a response rate of 40.7%. Excluding missing data for the outcome variables (*n* = 5870) yielded 67,699 unique participants, representing a response rate of 37.5%.

**Figure 1:**
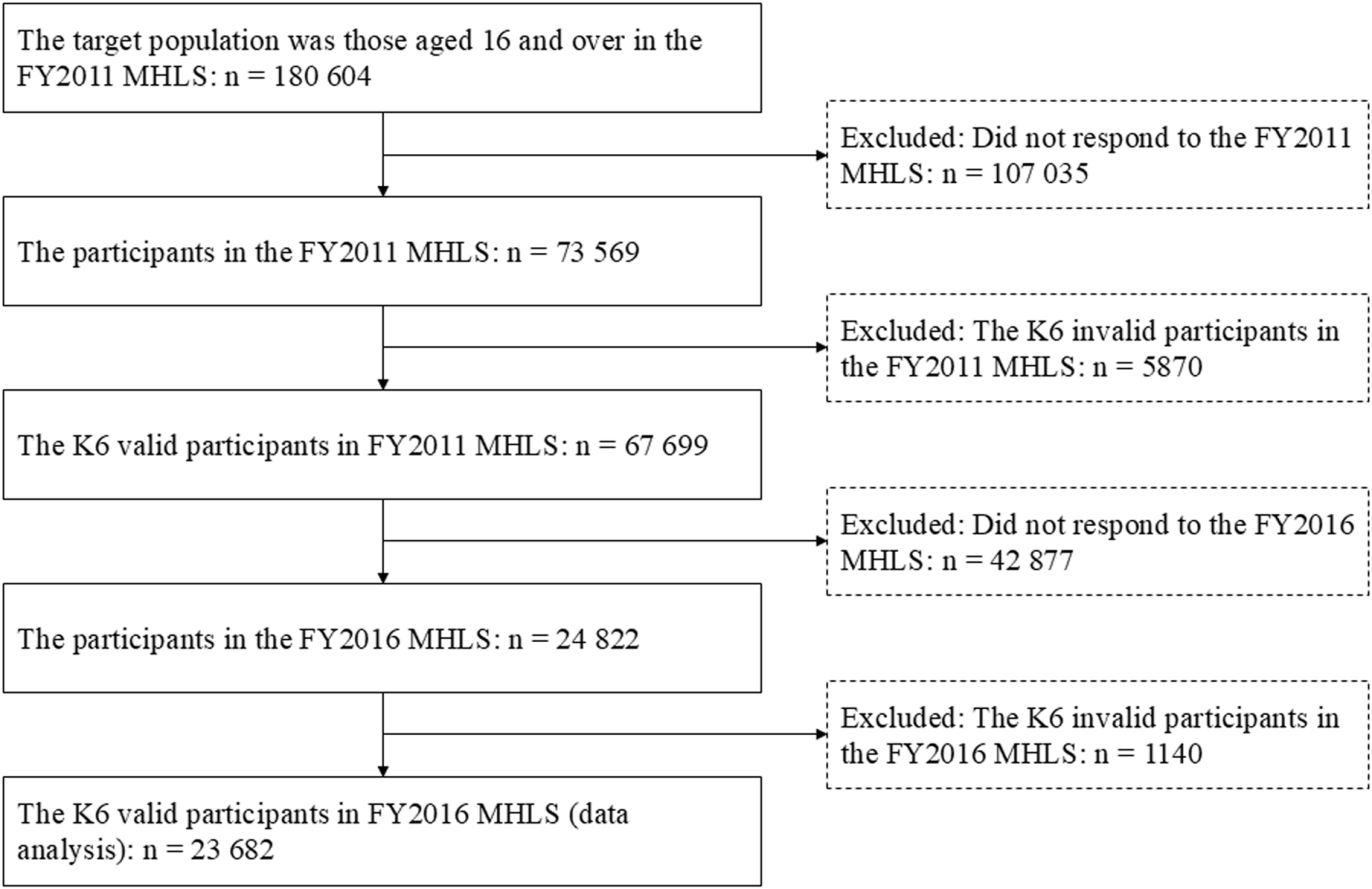
This flowchart outlines the participant selection and exclusion process. The target population for the FY2011 MHLS was 180,604, and 73,569 individuals responded to the survey. Of the 5870 individuals identified as invalid K6 cases in FY2011, 67,699 were included. Of the 42,877 nonrespondents in FY2016, 24,822 were included. Finally, of the 1140 participants with invalid K6 data in FY2016, 23,682 were included in the study. FY, fiscal year; K6, Kessler Psychological Distress Six‐Item Scale; MHLS, the Mental Health and Lifestyle Survey.

Of these, 24,822 individuals had also responded in FY2016, representing a response rate of 13.7%. After excluding participants with missing data on the outcome variables (*n* = 1140), 23,682 participants were included in the analysis, yielding a response rate of 13.1%. Only those who had responded to the K6 in both FY2011 and FY2016 were included in the final data analysis. A flowchart of the study is presented in Figure 1.

### Measures

#### Psychological distress

The outcome variable was nonspecific psychological distress, as measured using the K6.^13^ The K6 comprised six items occurring over the past 30 days, rated on a 5‐point Likert‐type scale ranging from 0 (none of the time) to 4 (all of the time), with a total range of 0–24. Higher scores indicated greater psychological distress. The Japanese version of the K6 used in this study has been validated.^25^, ^26^ Based on screening accuracy, previous studies^13^, ^27^ and the MHLS utilized a cut‐off score of ≥ 13 to indicate severe psychological distress.^21^, ^28^, ^29^, ^30^ Therefore, in the present study, we used total K6 scores, with individuals scoring ≥ 13 and <13 categorized into the psychological distress and control groups, respectively.

#### Background characteristics

Regarding background characteristics, the confounding variables were sex (male or female) and a history of diagnosed mental illness (no or yes). Youth aged 16–24 years are at a higher risk of mental illness than adults generally,^16^, ^17^ and the development of mental illness at this age negatively affects prognosis and functioning.^18^, ^19^ Furthermore, the MHLS revealed that a higher percentage of younger people experience severe psychological distress than those aged ≥ 65 years.^3^ Therefore, in the present study, the age subcohorts were divided into 16–24‐, 25–64‐, and ≥ 65‐year‐olds in FY2011.

#### Disaster‐related factors

Regarding disaster‐related factors, the explanatory variables included the place of residence, as in or out of the Fukushima Prefecture; experience of a tsunami (no or yes); experience of a nuclear reactor accident, which was defined as hearing a hydrogen explosion to limit the traumatic nature of the exposure (no or yes); house damage, which included no damage, partial damage, partial collapse, partial yet extensive collapse, or total collapse; and bereavement (no or yes). In this study, bereavement was defined as the loss of close relatives, friends, and significant others due to the earthquake, and based on the respondent's relationship to the deceased, with the deceased being a parent, spouse, child, sibling, friend, or other. Responses to the question, “Did you lose someone important or close to you in the disaster?” were assessed.

#### Health characteristics

Regarding health characteristics, the explanatory variables included subjective health status, sleep insufficiency, exercise, risk perception of genetic effects due to radiation, and PTSD symptoms. Subjective health status was measured using a 5‐point Likert‐type scale as follows: 1 = very good, 2 = good, 3 = average, 4 = poor, and 5 = very poor. Sleep insufficiency during the past month was measured using a 4‐point Likert‐type scale as follows: 1 = satisfied, 2 = slightly unsatisfied, 3 = quite unsatisfied, and 4 = very unsatisfied (did not sleep at all). However, this question did not include an objective measure of sleep duration. Exercise frequency was measured on a 4‐point Likert‐type scale as follows: 1 = almost every day; 2 = 2 to 4 times a week; 3 = once a week; and 4 = seldom or never.

We measured the risk perception of genetic effects due to radiation, using the following question: “What do you think is the likelihood that the health of your unborn/future children and grandchildren will be affected as a result of your current level of radiation exposure?” This question was translated into Japanese and subsequently back‐translated into English, after which it was modified in discussion with the questionnaire authors.^31^ The participants were requested to answer the questions on a 4‐point Likert scale as follows: 1 = very unlikely, 2 = unlikely, 3 = likely, and 4 = very likely.

PTSD symptoms were measured using the PTSD Checklist–Specific (PCL–S), based on the Diagnostic and Statistical Manual of Mental Disorders, Fourth Edition.^32^, ^33^ The PCL–S comprises 17 items related to PTSD symptoms experienced during the past month rated on a 5‐point Likert‐type scale ranging from 1 (not at all) to 5 (extremely), with a total range of 17–85. In this study, the specific traumatic experiences measured by the PCL–S included the GEJE, tsunami, and nuclear reactor accident. Higher scores indicated the presence of greater PTSD symptoms. The Japanese version of the PCL–S used in this study has been validated.^34^ Based on the screening and diagnostic accuracy thereof, previous studies^32^, ^33^, ^35^ and the MHLS have utilized a cut‐off score of ≥ 44 to indicate probable PTSD.^29^, ^36^, ^37^ Therefore, in the present study, we used the total PCL–S scores, whereby participants with scores of ≥ 44 and < 44 were categorized into probable PTSD and control groups, respectively. Although PTSD symptoms and psychological distress may share some variance, this study treated them as independent constructs and estimated the specific contribution of PTSD symptoms.

### Statistical analysis

We conducted *χ* ^2^ tests to compare the proportions of background, health characteristics, and disaster‐related factors in FY2011 between the psychological distress and control groups. We then conducted multiple logistic regression analyses to examine the association between age subcohorts in FY2011 and psychological distress in FY2016. We estimated odds ratios (ORs) and 95% confidence intervals (CIs) for unadjusted (Model 1) and adjusted models (Model 2). Model 2 controlled for sex, history of diagnosed mental illness, and psychological distress in FY2011, based on prior evidence.^21^, ^37^ We further conducted univariate and multivariate logistic regression analyses to examine associations of baseline background, health characteristics, and disaster‐related factors with psychological distress in FY2016. These analyses were stratified by age subcohort. Model 1 adjusted for background and health characteristics, including radiation risk perception, and Model 2 additionally adjusted for disaster‐related factors, consistent with previous studies.^38^, ^39^, ^40^ We handled missing data in explanatory variables using multiple imputation with 50 datasets. Imputation used a Markov chain Monte Carlo method based on a multivariate normal model under the missing‐at‐random assumption. We did not impute the outcome variable (K6) because cases with missing values were excluded per the study population definition. Therefore, only explanatory variables with missing data were imputed. All analyses were performed using SPSS Statistics, version 29 (IBM Corp.). Statistical significance was set at *p* < 0.05.

## RESULTS

### Sociodemographic information of the study population

Table 1 presents the sociodemographic characteristics of the study population. Of the 23,682 participants, 1650 (7.0%) and 22,032 (93.0%) were categorized into the psychological distress and control groups, respectively. At the FY2011 time point, 711 (3.0%), 13,756 (58.1%), and 9215 (38.9%) participants were aged 16–24, 25–64, and ≥ 65 years, respectively.

**Table 1:** Sociodemographic information of the study population.

		Variable in FY2016						
				K6 score < 13		K6 score ≥ 13		
Variable in FY2011		Total	(%)	*n*	(%)	*n*	(%)	*p*
No. of participants		23,682	‐	22,032	(93.0)	1650	(7.0)	
*Background characteristics*								
Sex								
Male		10574	(44.6)	9874	(44.8)	700	(42.4)	0.059
Female		13108	(55.4)	12158	(55.2)	950	(57.6)	
History of diagnosed mental illness								
No		21818	(94.8)	20542	(95.8)	1276	(81.8)	<0.001
Yes		1187	(5.2)	905	(4.2)	282	(18.1)	
*Disaster‐related factors*								
Place of residence								
In the Fukushima prefecture		19232	(81.2)	17959	(81.5)	1273	(77.2)	<0.001
Out of the Fukushima prefecture		4450	(18.8)	4073	(18.5)	377	(22.8)	
Experienced the tsunami								
No		18632	(78.7)	17414	(79.0)	1218	(73.8)	<0.001
Yes		5050	(21.3)	4618	(21.0)	432	(26.2)	
Experienced the nuclear reactor accident (explosion heard)								
No		10456	(44.2)	9924	(45.0)	532	(32.2)	<0.001
Yes		13226	(55.8)	12108	(55.0)	1118	(67.8)	
House damage								
No damage		5557	(25.0)	5272	(25.5)	285	(18.8)	<0.001
Partial damage		12764	(57.5)	11890	(57.5)	874	(57.7)	
Partial collapse		1802	(8.1)	1638	(7.9)	164	(10.8)	
Partial yet extensive collapse		706	(3.2)	631	(3.1)	75	(4.9)	
Total collapse		1357	(6.1)	1239	(6.0)	118	(7.8)	
Bereavement								
Yes		4805	(20.7)	4369	(20.2)	436	(27.2)	<0.001
No		18444	(79.3)	17275	(79.8)	1169	(72.8)	
*Health characteristics*								
Subjective health status								
Very good		788	(3.4)	772	(3.6)	16	(1.0)	<.001
Good		3088	(13.3)	3008	(13.9)	80	(5.0)	
Normal		14993	(64.5)	14289	(66.1)	704	(43.6)	
Poor		3988	(17.2)	3304	(15.3)	684	(42.4)	
Very poor		377	(1.6)	246	(1.1)	131	(8.1)	
Sleep insufficiency								
Satisfied		6842	(34.7)	6657	(36.3)	185	(13.5)	<0.001
Slightly dissatisfied		9000	(45.7)	8510	(46.4)	490	(35.8)	
Quite dissatisfied		3008	(15.3)	2569	(14.0)	439	(32.1)	
Very dissatisfied/could not sleep at all		841	(4.3)	587	(3.2)	254	(18.6)	
Exercise frequency								
Every day		3930	(16.9)	3702	(17.1)	228	(14.1)	0.002
2–4 times a week		5386	(23.1)	5018	(23.1)	368	(22.7)	
Once a week		3283	(14.1)	3068	(14.1)	215	(13.3)	
None		10721	(46.0)	9911	(45.7)	810	(50.0)	
Risk perception of genetic effects due to radiation								
Very unlikely		3621	(15.9)	3495	(16.5)	126	(8.0)	<0.001
Unlikely		5756	(25.4)	5508	(26.1)	248	(15.7)	
Likely		5722	(25.2)	5365	(25.4)	357	(22.6)	
Very likely		7604	(33.5)	6752	(32.0)	852	(53.8)	
Psychological distress								
K6 score < 13		20389	(86.1)	19630	(89.1)	759	(46.0)	<0.001
K6 score ≥ 13		3293	(13.9)	2402	(10.9)	891	(54.0)	
PTSD symptoms								
PCL–S score < 44		18283	(78.9)	17681	(82.0)	602	(37.4)	<0.001
PCL–S score ≥ 44		4883	(21.1)	3876	(18.0)	1007	(62.6)	

*Note*: *p* represents the chi‐square test. This table presents the sociodemographic information of the study population. Of the 23,682 participants, 1650 (7.0%) and 22 032 (93.0%) were categorized into the psychological distress and control groups, respectively, in FY2016. For psychological distress in FY2016, *χ* ^2^ tests were used to compare the proportions of background and health characteristics and of disaster‐related factors with those in FY2011.

Abbreviations: FY, fiscal year; K6, Kessler Psychological Distress Six‐Item Scale; PCL–S, Post‐traumatic Stress Disorder Checklist–Specific; PTSD, Post‐traumatic stress disorder.

We found the psychological distress group to be associated with a history of diagnosed mental illness, place of residence, experienced the tsunami, experienced the nuclear reactor accident (explosion heard), house damage, bereavement, subjective health status, sleep insufficiency, exercise frequency, the risk perception of genetic effects due to radiation, psychological distress in FY2011, and PTSD symptoms (*p* < 0.05).

### Correlations between age subcohorts and psychological distress

Table 2 presents the cross‐tabulation of the age subcohorts in FY2011 and psychological distress in FY2011 and FY2016. The results of the *χ* ^2^ test revealed that the correlation between the age subcohorts and the proportion of participants with psychological distress in FY2011 was not significant (*p* = 0.275). However, the results also revealed that the correlation between the age subcohorts and the proportion of participants with psychological distress in FY2016 was significant (*p* = 0.011). In FY2016, the proportion of participants with severe psychological distress was highest in the 16–24 age group (8.9%), followed by the ≥ 65 age group (7.4%) and the 25–64 age group (6.6%).

**Table 2:** Cross tabulation of age groups and psychological distress.

	Variable in FY2011				
	K6 < 13		K6 ≥ 13		
Variable in FY2011	*n*	%	*n*	%	*p*
16–24 years old	625	(87.9)	86	(12.1)	0.275
25–64 years old	11,855	(86.2)	1901	(13.8)	
≥65 years old	7909	(85.8)	1306	(14.2)	

	Variable in FY2016				
	K6 < 13		K6 ≥ 13		
Variable in FY2011	*n*	%	*n*	%	*p*
16–24 years old	648	(91.1)	63	(8.9)	0.011
25–64 years old	12,848	(93.4)	908	(6.6)	
≥65 years old	8536	(92.6)	679	(7.4)	

*Note*: This table presents a cross‐tabulation of age sub‐cohorts (i.e., 16–24, 25–64, and ≥ 65 years) in FY2011 and of psychological distress in FY2011 and FY2016. *χ* ^2^ tests were used to compare the FY2011 age subcohorts and the proportions of participants with psychological distress in FY2011 and FY2016, respectively.

Abbreviations: FY, fiscal year; K6, Kessler Psychological Distress Six‐Item Scale.

Table 3 presents the results of multiple logistic regression analyses with explanatory and outcome variables. The explanatory variables comprised the age subcohorts and covariates, including sex, history of diagnosed mental illness, and psychological distress in FY2011. The outcome variable was defined as psychological distress in FY2016. The results revealed that, in the unadjusted model, ages 25–64 years were significantly and negatively correlated with severe psychological distress in FY2016 compared with ages ≥ 65 years (OR = 0.888, 95% CI: 0.801–0.985, *p* = 0.025); however, this association was not significant after adjustment for covariates (OR = 0.899, 95% CI: 0.805–1.003, *p* = .056). In the adjusted model, ages 16–24 years were significantly and positively correlated with severe psychological distress in FY2016 compared with ages ≥ 65 years (OR = 1.389, 95% CI: 1.041–1.855, *p* = 0.026).

**Table 3:** Multiple logistic regression analysis for severe psychological distress in the fiscal year (FY) 2016.

	Model 1			Model 2		
Variable in FY2011	OR	95% CI	*P*	OR	95% CI	*P*
16–24 years old (Ref: ≥65 years old)	1.222	[0.933, 1.601]	0.146	1.389	[1.041, 1.855]	0.026
25–64 years old (Ref: ≥65 years old)	0.888	[0.801, 0.985]	0.025	0.899	[0.805, 1.003]	0.056
Sex (Ref: Male)		‐	‐	0.904	[0.812, 1.007]	0.067
History of diagnosed mental illness (Ref: No)	‐	‐	‐	1.167	[0.928, 1.467]	0.184
Psychological distress (Ref: K6 score < 13)	‐	‐	‐	9.464	[8.472, 10.571]	<0.001

*Note*: This table presents multiple logistic regression analyses, using the FY2011 age sub‐cohorts and FY2016 psychological distress. The explanatory variables included the age sub‐cohorts and covariates, including sex, history of diagnosed mental illness, and psychological distress in FY2011. The outcome variable was defined as psychological distress in FY2016. Model 1 was unadjusted, and Model 2 was adjusted for sex, history of diagnosed mental illness, and baseline psychological distress. Model 1: OR was unadjusted. Model 2: OR was adjusted for sex, history of diagnosed mental illness, and baseline psychological distress

Abbreviations: CI, confidence interval; K6, Kessler Psychological Distress Six‐Item Scale; OR, odds ratio.

### Correlation of background and health characteristics and disaster‐related factors with psychological distress

Supporting Information S1: Table S1 presents the results of the univariate logistic regression analyses stratified by the age subcohorts in FY2011, with background and health characteristics and disaster‐related factors in FY2011 as explanatory variables and psychological distress in FY2016 as the outcome variable. The results revealed that among those aged 16–24 years, subjective health status, sleep insufficiency, risk perception of genetic effects due to radiation, baseline psychological distress, and PTSD symptoms were significantly correlated with severe psychological distress in FY2016 (*p* < 0.05). In those aged 25–64 years, all explanatory variables, excluding sex, history of diagnosed mental illness, and exercise frequency, were significantly correlated with severe psychological distress in FY2016 (*p* < 0.05). Among those aged ≥ 65 years, all explanatory variables, excluding history of diagnosed mental illness, were significantly correlated with severe psychological distress in FY2016 (*p* < 0.001).

Table 4 presents the results of multiple logistic regression analyses stratified by the age subcohorts in FY2011, with background and health characteristics and disaster‐related factors in FY2011 as the explanatory variables and psychological distress in FY2016 as the outcome variable. All results, including Model 1 and the variance inflation factor (VIF), are presented in Supporting Information S1: Table S2. Specifically, the VIFs were < 5, indicating no multicollinearity. The results of Model 2 revealed that among those aged 16–24 years, baseline psychological distress (OR = 2.977, 95% CI: 1.323–6.701, *p* = 0.008), PTSD symptoms (OR = 3.078; 95% CI: 1.383–6.852; *p* = 0.006), and bereavement (OR = 4.234, 95% CI: 1.012–19.678, *p* = 0.046) were significantly correlated with severe psychological distress in FY2016. Among those aged 25–64 years, sex, subjective health status, sleep insufficiency, risk perception of genetic effects due to radiation, baseline psychological distress, and PTSD symptoms were significantly correlated with severe psychological distress in FY2016 (*p* < 0.05). Among those aged ≥ 65 years, subjective health status, sleep insufficiency, risk perception of genetic effects due to radiation, baseline psychological distress, PTSD symptoms, and place of residence were significantly correlated with severe psychological distress in FY2016 (*p* < 0.05).

**Table 4:** Multiple logistic analysis for severe psychological distress in FY2016, by age sub‐cohorts in FY2011.

	16–24 years old			25–64 years old		
	Model 2			Model 2		
Variable in FY2011	OR	95% CI	*p*	OR	95% CI	*p*
Sex (Ref: Male)	0.753	[0.404, 1.403]	0.371	0.768	[0.660, 0.893]	<0.001
History of diagnosed mental illness (Ref: No)	1.046	[0.590, 1.854]	0.879	1.143	[0.928, 1.408]	0.206
Subjective health status	1.026	[0.710, 1.482]	0.890	1.464	[1.294, 1.655]	<0.001
Sleep insufficiency	1.302	[0.851, 1.990]	0.224	1.410	[1.273, 1.562]	<0.001
Exercise frequency	1.082	[0.833, 1.406]	0.555	1.033	[0.962, 1.110]	0.370
Risk perception of genetic effects due to radiation	1.035	[0.781, 1.371]	0.813	1.091	[1.009, 1.179]	0.028
Psychological distress (Ref: K6 score < 13)	2.977	[1.323, 6.701]	0.008	3.210	[2.668, 3.861]	<0.001
PTSD symptoms (Ref: PCL–S score < 44)	3.078	[1.383, 6.852]	0.006	2.674	[2.211, 3.234]	<0.001
Place of residence (Ref: In the Fukushima prefecture)	1.061	[0.561, 2.006]	0.856	1.023	[0.859, 1.217]	0.801
Experienced the tsunami (Ref: no)	0.652	[0.281, 1.517]	0.321	0.968	[0.805, 1.163]	0.728
Experienced the nuclear reactor accident (explosion heard) (Ref: No)	1.086	[0.604, 1.952]	0.784	1.141	[0.974, 1.336]	0.103
House damage (Ref: No)	1.129	[0.848, 1.502]	0.406	0.991	[0.919, 1.068]	0.810
Bereavement (Ref: No)	4.234	[1.012, 19.678]	0.046	0.929	[0.759, 1.137]	0.475

	≥ 65 years old		
	Model 2		
Variable in FY2011	OR	95% CI	*p*
Sex (Ref: Male)	0.870	[0.731, 1.036]	0.119
History of diagnosed mental illness (Ref: No)	1.069	[0.911, 1.253]	0.413
Subjective health status	1.741	[1.502, 2.017]	<0.001
Sleep insufficiency	1.264	[1.124, 1.421]	<0.001
Exercise frequency	1.074	[0.994, 1.161]	0.070
Risk perception of genetic effects due to radiation	1.143	[1.038, 1.257]	0.006
Psychological distress (Ref: K6 score < 13)	3.355	[2.708, 4.157]	<0.001
PTSD symptoms (Ref: PCL–S score < 44)	2.071	[1.651, 2.597]	<0.001
Place of residence (Ref: In the Fukushima prefecture)	1.329	[1.073, 1.647]	0.009
Experienced the tsunami (Ref: no)	1.013	[0.827, 1.240]	0.903
Experienced the nuclear reactor accident (explosion heard) (Ref: No)	1.050	[0.865, 1.275]	0.624
House damage (Ref: No)	1.088	[0.993, 1.193]	0.071
Bereavement (Ref: No)	1.032	[0.931, 1.144]	0.545

*Note*: This table presents multiple logistic regression analyses stratified by age sub‐cohorts in FY2011, with background, health characteristics, and disaster‐related factors in FY2011 as the explanatory variables, and psychological distress in FY2016 as the outcome. Model 1 was adjusted for background and health characteristics in FY2011, while Model 2 was adjusted for those factors plus disaster‐related factors in FY2011. This table shows only the results for Model 2, which does not include variance inflation factor (VIF) values; Supporting Information S1: Table S2 presents the complete results, including Model 1 and the VIF values. Model 2: OR was adjusted for background and health characteristics and disaster‐related factors.

Abbreviations: CI, confidence interval; FY, fiscal year; K6, Kessler Psychological Distress Six‐Item Scale; OR, odds ratio; PCL–S, Post‐traumatic Stress Disorder Checklist–Specific Version; PTSD, post‐traumatic stress disorder.

## DISCUSSION

This study examined longitudinal risk factors for psychological distress among youth after the GEJE and nuclear disaster, revealing several key findings.

First, after controlling for confounders, such as baseline psychological distress, ages 16–24 years at baseline were positively correlated with severe psychological distress at the 5‐year follow‐up, when compared with ages ≥ 65 years. Several previous studies have revealed that adolescents and youth aged 16–24 years are at a higher risk of developing mental illness than other age groups^16^, ^17^ and that mental health problems in this age group are associated with poor prognosis and functioning.^18^, ^19^ The GEJE and nuclear disaster caused large‐scale damage due to natural and nuclear hazards. Residents were forced to undergo long‐term evacuation and protection from radiation exposure.^1^ Those aged 16–24 years are likely to have experienced substantial changes in their school and work situations, as well as disruptions in peer relationships associated with long‐term evacuation, given the profound social changes caused by forced displacement following the disaster.^23^, ^41^, ^42^ As adolescents need to establish self‐identity and social roles during their school years, these drastic changes could have had a worse impact, resulting in poor mental health 5 years after the disaster.

Second, after controlling for a baseline history of diagnosed mental illness and psychological distress, PTSD symptoms and bereavement were correlated with severe psychological distress at the 5‐year follow‐up in those aged 16–24 years. Previous studies have shown that traumatic experiences at a young age can negatively impact physical and mental development, resulting in future poor mental health and functional impairment.^43^, ^44^, ^45^ Moreover, prospective studies have revealed that trauma exposure and PTSD are predictive of higher rates of psychopathology, including depression; substance use disorders; risk events, such as suicide attempts; and functional impairment in youth.^45^, ^46^ A cross‐sectional study conducted after the Fukushima disaster revealed that traumatic experiences were associated with severe psychological distress among adolescents.^21^ Furthermore, bereavement is associated with various mental health outcomes, including PTSD^47^, ^48^, ^49^ and grief reactions to the loss of significant others are likely to persist over a long period. For example, those who experienced bereavement after the Fukushima disaster were found to be at a higher risk of severe PTSD symptoms, particularly when the deceased was a spouse, child, or friend.^36^ Given these findings, it is possible that grief reactions among young people following the loss of a loved one are associated with long‐term poor mental health.

The results of this study suggested that risk factors other than PTSD symptoms varied by the age subcohort. In particular, for ages 25–64 years, female sex, poor subjective health status, sleep insufficiency, risk perception of genetic effects due to radiation, and PTSD symptoms were correlated with severe psychological distress at the 5‐year follow‐up. Generally, those aged 25–64 years are more likely to be employed and to have families, and they often experience significant life transitions that shape their social and community engagement. A previous study of middle‐aged adults in Japan found that social networks characterized by well‐established friendship ties, participation in hobbies, neighborly ties, and involvement in community activities were negatively associated with psychological distress.^50^ Conversely, for those aged 25–64 years, health characteristics, such as poor subjective health status and sleep insufficiency, were correlated with long‐term poor mental health, whereas disaster‐related variables were not associated with such deterioration. One possible explanation is that disaster‐related factors alone may not sufficiently explain long‐term psychological distress. Rather, their impact may depend on interactions with individual health conditions, such as subjective health status and sleep quality. In future research, examining such interactions may help clarify vulnerable groups associated with long‐term poor mental health.

In sum, the findings indicate that the factors associated with severe psychological distress differed across age subcohorts at the 5‐year follow‐up. This study focused on examining psychological distress and its associated factors among youth. Future studies should also examine the risk factors correlated with psychological distress in middle‐aged and older adults. Furthermore, our study results showed that baseline PTSD symptoms were correlated with severe psychological distress at the 5‐year follow‐up, across all age subcohorts. Therefore, the traumatic event experienced by numerous people during the GEJE and nuclear disaster may have considerable mental health effects across generations.

## LIMITATIONS

This study has several limitations. First, the study included only those who responded to the MHLS in FY2011 and FY2016. As 40.7% and 20.4% responded to the surveys in FY2011 and FY2016, respectively,^3^ the generalizability of the study's findings is limited. This low response rate is likely attributable, at least in part, to the disaster‐specific social disruption that persisted from FY2011 to FY2016. Specifically, prolonged evacuation orders imposed on residents of affected municipalities and frequent relocations made it difficult to maintain long‐term contact with participants. Young people aged 16–24 may have been particularly susceptible to these disruptions, given that this age group commonly undergoes major life transitions—such as relocations for educational or employment purposes—that inherently involve geographic and social instability. In addition, the MHLS comprises 105 items in FY2011 and 68 in FY2016, which may have increased the burden on respondents and consequently lowered the response rate. An interview study investigating differences between MHLS respondents and nonrespondents showed that nonrespondents had significantly higher rates of psychological distress than respondents.^51^ Although this finding pertains to nonresponse during FY2011–2013, it suggests that nonparticipation in the survey was systematically associated with greater psychological distress within this cohort. Therefore, the findings of this study may underestimate the true impact on psychological distress in youth, who may have been more vulnerable to the instability of residence associated with evacuation.

Second, the sample size of the 16–24 age group in this study was small (3.0% of the total). As this study focused on mental health following large‐scale accidental disasters, recruiting participants based on sample size calculations was not feasible. In addition, separating the effects of the Fukushima disaster from the natural life course of psychological distress was difficult in this study design. Third, socioeconomic factors have been reported to influence mental health,^52^ but this study was unable to examine these relationships. Finally, the outcome period from baseline was set at a 5‐year follow‐up, and only data from these two time points were used.

## CONCLUSIONS

This study suggested that, for youth aged 16–24 years, experiencing the GEJE and the nuclear disaster was associated with long‐term poor mental health. In addition, baseline PTSD symptoms and bereavement may be associated with long‐term poor mental health. Although more than 15 years have passed since this disaster, over 26,000 people were still forced to evacuate.^4^ Therefore, it is important to clarify further factors associated with mental health outcomes following the GEJE and the Fukushima Daiichi Nuclear Power Plant accident. Moreover, developing effective psychosocial interventions that account for age‐specific risk factors is essential.

## AUTHOR CONTRIBUTIONS

**Hideki Sato**: Conceptualization; data curation; formal analysis; methodology; writing—original draft. **Masaharu Maeda**: Conceptualization; data curation; funding acquisition; investigation; project administration; resources; supervision; writing – review and editing. **Rie Mizuki**: Conceptualization; data curation; formal analysis; methodology; writing – review and editing. **Naoko Horikoshi**: Data curation; investigation; writing – review and editing. **Shuntaro Itagaki**: Data curation; writing – review and editing. **Itaru Miura**: Data curation; funding acquisition; investigation; project administration; resources; writing—review and editing. **Tetsuya Ohira**: Data curation; funding acquisition; investigation; project administration; resources; writing—review and editing. **Seiji Yasumura**: Data curation; funding acquisition; investigation; project administration; resources; writing—review and editing.

## CONFLICT OF INTEREST STATEMENT

The authors declare no conflicts of interest.

## ETHICS APPROVAL STATEMENT

This study was approved by the Ethics Committee of the Fukushima Medical University (Approval number 2020‐239). Moreover, this study was conducted in accordance with the principles of the Declaration of Helsinki and the Committee on Publication Ethics.

## PATIENT CONSENT STATEMENT

All participants were informed of the purpose of this study and were told the following: (1) participation is entirely voluntary; (2) they may withdraw from the study at any time; and (3) they will not suffer any disadvantage if they decline to participate or withdraw from the study. Furthermore, the return of the questionnaire was considered to indicate the respondent's consent to participate in this survey.

## CLINICAL TRIAL REGISTRATION

Not applicable.

## Supporting information

Supporting File 1.

## Data Availability

The dataset analyzed in this study is not publicly available, as the data from the Fukushima Health Management Survey belong to the Fukushima Prefectural Government and are restricted to use within that organization.
