# Supplementary material for: Five‐year follow‐up of the risk factors for psychological distress in youth after the Great East Japan Earthquake and nuclear disaster: The Fukushima Health Management Survey
Source: PCN Rep. 2026 Jul 15;5(3):e70377. doi: 10.1002/pcn5.70377 (PMC13370328; doi:10.1002/pcn5.70377)
Supplement: Supplementary file 1 — Supporting File 1. [file PCN5-5-e70377-s001.docx]

| **Table S1: Univariate logistic analysis for severe psychological distress in FY2016, by age sub-cohorts in FY2011** | | | | | | | | | | | |
| --- | --- | --- | --- | --- | --- | --- | --- | --- | --- | --- | --- |
|  | 16–24 years old | | |  | 25–64 years old | | |  | ≥ 65 years old | | |
| Variable in FY2011 | OR | 95% CI | *P* |  | OR | 95% CI | *P* |  | OR | 95% CI | *P* |
| Sex (Ref: Male) | 1.151 | [0.661, 2.005] | .618 |  | 1.000 | [0.872, 1.146] | .997 |  | **1.261** | **[1.077, 1.476]** | **.004** |
| History of diagnosed mental illness ( Ref: No) | 1.405 | [0.715, 2.762] | .323 |  | 1.368 | [0.782, 2.392] | .269 |  | 1.282 | [0.793, 2.075] | .308 |
| Subjective health status | **1.777** | **[1.280, 2.466]** | **<.001** |  | **3.257** | **[2.939, 3.609]** | **<.001** |  | **3.347** | **[2.956, 3.789]** | **<.001** |
| Sleep insufficiency | **2.271** | **[1.631, 3.162]** | **<.001** |  | **2.648** | **[2.439, 2.876]** | **<.001** |  | **2.329** | **[2.121, 2.558]** | **<.001** |
| Exercise frequency | 1.141 | [0.895, 1.454] | .288 |  | 1.066 | [0.999, 1.138] | .054 |  | **1.165** | **[1.086, 1.248]** | **<.001** |
| Risk perception of genetic effects due to radiation | **1.332** | **[1.035, 1.714]** | **.026** |  | **1.604** | **[1.494, 1.722]** | **<.001** |  | **1.625** | **[1.490, 1.773]** | **<.001** |
| Psychological distress (Ref: K6 score < 13) | **7.487** | **[4.250, 13.190]** | **<.001** |  | **10.066** | **[8.733, 11.602]** | **<.001** |  | **9.25** | **[7.840, 10.912]** | **<.001** |
| PTSD symptoms (Ref: PCL–S score < 44) | **7.389** | **[4.236, 12.889]** | **<.001** |  | **8.481** | **[7.356, 9.777]** | **<.001** |  | **6.611** | **[5.586, 7.823]** | **<.001** |
| Place of residence (Ref: In the Fukushima prefecture) | 1.220 | [0.686, 2.168] | .498 |  | **1.187** | **[1.013, 1.391]** | **.034** |  | **1.573** | **[1.295, 1.910]** | **<.001** |
| Experienced the tsunami (Ref: No) | .893 | [0.441, 1.809] | .754 |  | **1.311** | **[1.121, 1.534]** | **<.001** |  | **1.404** | **[1.180, 1.669]** | **<.001** |
| Experienced the nuclear reactor accident (explosion heard) (Ref: No) | 1.428 | [0.850, 2.397] | .178 |  | **1.79** | **[1.554, 2.061]** | **<.001** |  | **1.658** | **[1.395, 1.971]** | **<.001** |
| House damage (Ref: No) | 1.047 | [0.827, 1.326] | .700 |  | **1.126** | **[1.058, 1.199]** | **<.001** |  | **1.298** | **[1.205, 1.398]** | **<.001** |
| Bereavement (Ref: No) | 2.568 | [0.911, 7.239] | .074 |  | **0.634** | **[0.544, 0.739]** | **<.001** |  | 0.693 | [0.580, 0.828] | **<.001** |
| Abbreviations: FY = fiscal year; K6 = Kessler Psychological Distress Six-Item Scale; PCL–S = Post-traumatic Stress Disorder Checklist–Specific Version; OR = odds ratio; CI = confidence interval; PTSD = Post-traumatic stress disorder. | | | | | | | | | | | |

This table presents univariate logistic regression analyses stratified by age sub-cohorts in FY2011, with background and health characteristics and disaster-related factors in FY2011 as explanatory variables and psychological distress in FY2016 as the outcome variable.

| **Table S2: Multiple logistic analysis for severe psychological distress in FY2016, by age sub-cohorts in FY2011** | | | | | | | | | | | | | | | | | |
| --- | --- | --- | --- | --- | --- | --- | --- | --- | --- | --- | --- | --- | --- | --- | --- | --- | --- |
|  | 16–24 years old | | | | | | | |  | 25–64 years old | | | | | | | |
|  | Model 1 | | | | Model 2 | | | |  | Model 1 | | | | Model 2 | | | |
| Variable in FY2011 | OR | 95% CI | *P* | VIF | OR | 95% CI | *P* | VIF |  | OR | 95% CI | *P* | VIF | OR | 95% CI | *P* | VIF |
| Sex (Ref: Male) | .771 | [0.415, 1.433] | .411 | 1.064 | 0.753 | [0.404, 1.403] | .371 | 1.070 |  | **0.766** | **[0.659, 0.890]** | **<.001** | 1.016 | **0.768** | **[0.660, 0.893]** | **<.001** | 1.036 |
| History of diagnosed mental illness (Ref: No) | 1.060 | [0.603, 1.863] | .840 | 1.188 | 1.046 | [0.590, 1.854] | .879 | 1.161 |  | 1.143 | [0.929, 1.406] | .205 | 1.053 | 1.143 | [0.928, 1.408] | .206 | 1.053 |
| Subjective health status | 1.042 | [0.725, 1.496] | .824 | 1.247 | 1.026 | [0.710, 1.482] | .890 | 1.225 |  | **1.471** | **[1.301, 1.662]** | **<.001** | 1.291 | **1.464** | **[1.294, 1.655]** | **<.001** | 1.288 |
| Sleep insufficiency | 1.266 | [0.834, 1.922] | .268 | 1.373 | 1.302 | [0.851, 1.990] | .224 | 1.366 |  | **1.415** | **[1.278, 1.567]** | **<.001** | 1.347 | **1.410** | **[1.273, 1.562]** | **<.001** | 1.341 |
| Exercise frequency | 1.090 | [0.841, 1.412] | .516 | 1.040 | 1.082 | [0.833, 1.406] | .555 | 1.049 |  | 1.031 | [0.960, 1.107] | .402 | 1.010 | 1.033 | [0.962, 1.110] | .370 | 1.014 |
| Risk perception of genetic effects due to radiation | 1.044 | [0.790, 1.380] | .761 | 1.059 | 1.035 | [0.781, 1.371] | .813 | 1.068 |  | **1.095** | **[1.014, 1.184]** | **.021** | 1.115 | **1.091** | **[1.009, 1.179]** | **.028** | 1.129 |
| Psychological distress (Ref: K6 score < 13) | **2.927** | **[1.318, 6.498]** | **.008** | 1.690 | **2.977** | **[1.323, 6.701]** | **.008** | 1.720 |  | **3.214** | **[2.672, 3.867]** | **<.001** | 1.506 | **3.210** | **[2.668, 3.861]** | **<.001** | 1.513 |
| PTSD symptoms (Ref: PCL–S score < 44) | **3.030** | **[1.374, 6.682]** | **.006** | 1.682 | **3.078** | **[1.383, 6.852]** | **.006** | 1.724 |  | **2.707** | **[2.242, 3.269]** | **<.001** | 1.540 | **2.674** | **[2.211, 3.234]** | **<.001** | 1.560 |
| Place of residence (Ref: In the Fukushima prefecture) | - | - | - | - | 1.061 | [0.561, 2.006] | .856 | 1.031 |  | - | - | - | - | 1.023 | [0.859, 1.217] | .801 | 1.008 |
| Experienced the tsunami (Ref: no) | - | - | - | - | 0.652 | [0.281, 1.517] | .321 | 1.231 |  | - | - | - | - | 0.968 | [0.805, 1.163] | .728 | 1.189 |
| Experienced the nuclear reactor accident (explosion heard) (Ref: No) | - | - | - | - | 1.086 | [0.604, 1.952] | .784 | 1.124 |  | - | - | - | - | 1.141 | [0.974, 1.336] | .103 | 1.099 |
| House damage (Ref: No) | - | - | - | - | 1.129 | [0.848, 1.502] | .406 | 1.211 |  | - | - | - | - | 0.991 | [0.919, 1.068] | .810 | 1.132 |
| Bereavement (Ref: No) | - | - | - | - | **4.234** | **[1.012, 19.678]** | **.046** | 1.052 |  | - | - | - | - | 0.929 | [0.759, 1.137] | .475 | 1.075 |
|  | ≥ 65 years old | | | | | | | |  |  |  |  |  |  |  |  |  |
|  | Model 1 | | | | Model 2 | | | |  |  |  |  |  |  |  |  |  |
| Variable in FY2011 | OR | 95% CI | *P* | VIF | OR | 95% CI | *P* | VIF |  |  |  |  |  |  |  |  |  |
| Sex (Ref: Male) | 0.871 | [0.732, 1.036] | .119 | 1.032 | 0.870 | [0.731, 1.036] | .119 | 1.038 |  |  |  |  |  |  |  |  |  |
| History of diagnosed mental illness (Ref: No) | 1.070 | [0.913, 1.254] | .401 | 1.060 | 1.069 | [0.911, 1.253] | .413 | 1.056 |  |  |  |  |  |  |  |  |  |
| Subjective health status | **1.754** | **[1.514, 2.032]** | **<.001** | 1.277 | **1.741** | **[1.502, 2.017]** | **<.001** | 1.279 |  |  |  |  |  |  |  |  |  |
| Sleep insufficiency | **1.275** | **[1.135, 1.433]** | **<.001** | 1.339 | **1.264** | **[1.124, 1.421]** | **<.001** | 1.340 |  |  |  |  |  |  |  |  |  |
| Exercise frequency | 1.068 | [0.989, 1.154] | .093 | 1.030 | 1.074 | [0.994, 1.161] | .070 | 1.033 |  |  |  |  |  |  |  |  |  |
| Risk perception of genetic effects due to radiation | **1.149** | **[1.044, 1.264]** | **.004** | 1.102 | **1.143** | **[1.038, 1.257]** | **.006** | 1.124 |  |  |  |  |  |  |  |  |  |
| Psychological distress (Ref: K6 score < 13) | **3.353** | **[2.708, 4.152]** | **<.001** | 1.472 | **3.355** | **[2.708, 4.157]** | **<.001** | 1.482 |  |  |  |  |  |  |  |  |  |
| PTSD symptoms (Ref: PCL–S score < 44) | **2.129** | **[1.701, 2.666]** | **<.001** | 1.537 | **2.071** | **[1.651, 2.597]** | **<.001** | 1.582 |  |  |  |  |  |  |  |  |  |
| Place of residence (Ref: In the Fukushima prefecture) | - | - | - | - | **1.329** | **[1.073, 1.647]** | **.009** | 1.014 |  |  |  |  |  |  |  |  |  |
| Experienced the tsunami (Ref: no) | - | - | - | - | 1.013 | [0.827, 1.240] | .903 | 1.164 |  |  |  |  |  |  |  |  |  |
| Experienced the nuclear reactor accident (explosion heard) (Ref: No) | - | - | - | - | 1.050 | [0.865, 1.275] | .624 | 1.110 |  |  |  |  |  |  |  |  |  |
| House damage (Ref: No) | - | - | - | - | 1.088 | [0.993, 1.193] | .071 | 1.130 |  |  |  |  |  |  |  |  |  |
| Bereavement (Ref: No) | - | - | - | - | 1.032 | [0.931, 1.144] | .545 | 1.080 |  |  |  |  |  |  |  |  |  |
| Abbreviations: FY = fiscal year; K6 = Kessler Psychological Distress Six-Item Scale; PCL–S = Post-traumatic Stress Disorder Checklist–Specific Version; OR = odds ratio; CI = confidence interval; PTSD = Post-traumatic stress disorder; VIF = variance inflation factor. | | | | | | | | | | | | | | | | | |
| Model 1: OR was adjusted for background and health characteristics. | | | | | | | | | | | | | | | | | |
| Model 2: OR was adjusted for background and health characteristics and disaster-related factors. | | | | | | | | | | | | | | | | | |

This table presents multiple logistic regression analyses stratified by age sub-cohorts in FY2011, with background, health characteristics, and disaster-related factors in FY2011 as the explanatory variables, and psychological distress in FY2016 as the outcome. Model 1 was adjusted for background and health characteristics in FY2011, while Model 2 was adjusted for those factors plus disaster-related factors in FY2011. This table presents full results, including Model 1 and VIF values, which are not included in Table 4.
